# Genetic Evidence Links Gestational Diabetes Mellitus to Increased ER‐Negative Breast Cancer Risk Through a Mendelian Randomization Analysis

**DOI:** 10.1155/ije/7371037

**Published:** 2026-04-28

**Authors:** Wangwang Peng, Zhulan Huang, Min Zhang

**Affiliations:** ^1^ Department of Ultrasound, Maternity and Child Healthcare Hospital of Longgang District, Shenzhen City (Affiliated Shenzhen Women and Children′s Hospital (Longgang) of Shantou University Medical College), No. 6 Ailong Road Longgang District, Shenzhen, 518172, China

**Keywords:** breast cancer, causal inference, ER-negative breast cancer, gestational diabetes mellitus, Mendelian randomization

## Abstract

Observational studies reached conflicting results regarding the association between gestational diabetes mellitus (GDM) and breast cancer (BC), leaving the causal nature of this relationship unresolved. To clarify this, a Mendelian randomization (MR) study was performed. In brief, genetic instruments for GDM were obtained from the FinnGen project, and data for BC were obtained from previously published consortia. The primary MR analysis methods included the inverse‐variance weighted method, with MR‐Egger, weighted median, and weighted mode methods serving as validation. Heterogeneity, pleiotropy, and the influence of outliers were evaluated using mandatory sensitivity analyses. Additional sensitivity analyses included Steiger directionality test, radial MR analysis, and multivariable MR analysis. Genetically predicted GDM demonstrated a significant correlation with an increased risk of overall BC (OR = 1.17, 95% CI: 1.03–1.32, *p* = 0.01) and specifically with ER‐negative BC (OR = 1.18, 95% CI: 1.04–1.35, *p* = 0.01). No such association was observed for ER‐positive BC (*p* = 0.49). However, the causal effect of BC on GDM risk did not emerge in the reverse MR analysis. Comprehensive sensitivity analyses supported these findings. This study provides further evidence that GDM may be a risk factor for BC, particularly the ER‐negative subtype, suggesting that GDM may be incorporated into personalized risk assessment models.

## 1. Introduction

Breast cancer (BC) stands as the most prevalent malignancy in women and a primary public health concern, constituting roughly 30% of all new female cancer diagnoses. According to 2022 global data, an estimated 2.3 million women were diagnosed with BC (11.6% of all cancers), and the disease resulted in approximately 665,684 deaths, accounting for 6.9% of all cancer‐related mortality [1]. Although BC has a relatively favorable prognosis compared with other cancers and despite significant advancements in treatment, several challenges remain, with impacts on treatment efficacy, patient outcomes, quality of life, and survivorship [2, 3]. Notably, its incidence has increased by approximately 3% annually, driven by factors such as aging populations and lifestyle changes [4]. Although the factors predicting BC risk are well documented, the recognized factors fail to accurately predict BC risk [5]. The identification of novel risk factors for BC thus remains a clinical imperative, with the potential to strengthen early detection and prevention, guide the development of personalized treatment strategies, and improve the current standard of care for patients [6].

Gestational diabetes mellitus (GDM) refers to a distinct type of disease symptom first diagnosed during the mid‐to‐late stages of pregnancy in women without a pre‐existing history of overt diabetes [7, 8]. Clinically, the development of GDM during pregnancy has been linked to a heightened risk of preeclampsia, eclampsia, preterm birth, primary cesarean delivery, and metabolic syndrome [7]. Beyond the perinatal period, GDM is recognized for its potential long‐term health consequences, including an elevated risk for subsequent cancers, notably BC [9, 10]. Some reports have indicated that the risk of developing BC in women with GDM may surpass 7% [9–11]. Several biological mechanisms have been proposed as bridging GDM to BC, including hormonal imbalances, chronic metabolic dysfunction, and inflammation [12]. Still, many observational studies reported no associations between GDM and BC, as reviewed by Wang et al. [9]. Reasons can include the heterogeneity of BC that can display vast differences in biology among patients and the numerous confounders (e.g., age, reproductive history, exogenous hormone exposure, and radiation exposure) [5, 13]. Indeed, Park et al. [14] reported a substantial association between GDM and estrogen receptor (ER)–positive BC but only a slight association with ER‐negative BC. Nevertheless, despite the valuable insights provided by observational studies into the relationship between GDM and BC, these studies are constrained by potential confounding variables [15].

Mendelian randomization (MR) is a crucial analytical tool in modern clinical and statistical epidemiology that uses randomly allocated genetic variants as instrumental variables (IVs) to infer causal relationships through statistical testing [16]. Therefore, MR analysis could mitigate confounding, reverse causality, measurement errors, and biases, which are important limitations of traditional epidemiological studies [17–19]. This approach offers a significant analytical advantage in assessing causal associations between risk factors and health outcomes within observational data [16]. Previous MR studies have assessed many key risk or protective factors in BC, such as body mass index (BMI) and fasting insulin [20–22], but the role of GDM is still poorly defined.

Therefore, the study aimed to examine the causal relationship between GDM and BC. The results may reveal the mechanisms by which genetically predisposed GDM influences BC and contribute to new research directions and potential therapeutic targets.

## 2. Methods

### 2.1. Data Procurement and Processing

We first utilized summary‐level data from genome‐wide association studies (GWAS) for various BC subtypes, including overall BC (76,192 cases and 63,082 controls) [23], ER‐negative BC (14,135 cases and 58,126 controls) [24], and ER‐positive BC (69,501 cases and 105,974 controls) [23, 24]. Concurrently, data for GDM were sourced from the FinnGen R12 project (finn‐b‐Gest Diabetes), which comprised 5687 cases and 117,892 controls. The diagnosis of GDM in this cohort adhered to the World Health Organization criteria (a 75‐g oral glucose tolerance test with fasting, 1‐h, or 2‐h glucose levels greater than 5.1, 10.0, or 8.5 mmol/L, respectively). Detailed information on all datasets used is provided in Supporting Table S1.

**FIGURE 1:**
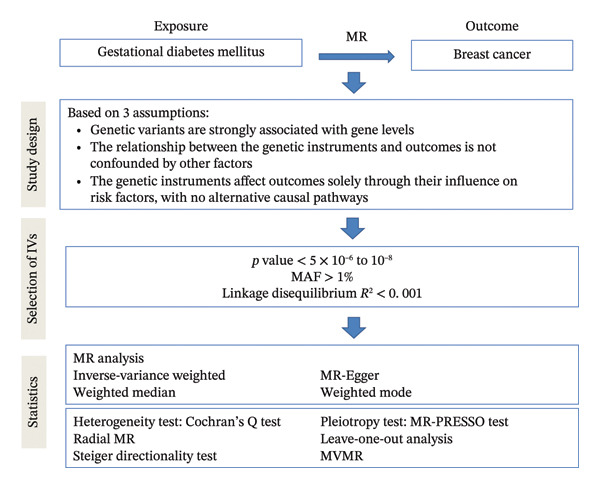
Study design. MR: Mendelian randomization; MR‐PRESSO: MR‐Egger and MR pleiotropy residual sum and outlier; MAF: minor allele frequency; GDM: gestational diabetes mellitus.

Subsequently, IVs were screened for the formal analysis. The selected genetic IVs were required to satisfy the three key MR assumptions [25–27]: relevance, independence, and exclusion restriction. A comprehensive overview of the study design is illustrated in Figure 1.

### 2.2. Selection

Specifically, the selection of IVs adhered to the following parameter criteria [28]. First, to ensure a sufficient number of statistically powerful SNPs, appropriate *P*‐value thresholds were established. For the forward analysis, SNPs linked to overall BC and the ER‐positive subtype were selected using the conventional criterion of *p* < 5 × 10^−8^ [29, 30]. Relaxing the threshold is sometimes necessary to ensure sufficient IVs when the more stringent threshold does not identify enough SNPs for MR analysis [31–33]. To secure an adequate number of IVs for GDM and for the ER‐negative subtype as exposure, the selection thresholds were *p* < 5 × 10^−7^ and *p* < 5 × 10^−6^, respectively. Furthermore, for subsequent analyses, only SNPs with a minor allele frequency (MAF) of 0.01 or greater were retained [34].

Second, to remove the influence of linkage disequilibrium (LD), clumping was performed using a strict threshold of *R* ^2^ < 0.001 within a 10,000‐kb window [35]. An F‐statistic > 10 was used to avoid weak instruments. When selected IVs were absent from the summary data, a proxy SNP with high LD (*R* ^2^ > 0.8) was identified as a replacement using the LDlink online platform (https://ldlink.nci.nih.gov/) [36]. Finally, a harmonization process was performed to align the effect alleles for exposure and outcome SNPs, removing any incompatible alleles and palindromic SNPs with intermediate frequencies.

### 2.3. MR Analysis

For formal MR analyses, the “TwoSampleMR” package in *R* Version 4.0.5 was the main tool. The primary analysis employed the inverse‐variance weighted (IVW) method to estimate the odds ratio (OR) and 95% confidence interval (CI), thereby assessing the causal relationship between exposure and outcome. It was achieved by calculating a weighted average of the effect size from each SNP, with the inverse variance serving as the weight [37, 38]. The findings were validated using the MR‐Egger [39], weighted median [40], and weighted model [41] methods; consistency between these results and the IVW estimate is considered indicative of a strong causal association. The MR‐Egger method accounts for horizontal pleiotropy by incorporating an intercept term, thereby enabling more accurate estimation of the causal effect. The weighted median method remains valid as long as at least 50% of the IVs are correctly specified, providing an additional layer of validation for the causal links between exposure and outcome.

### 2.4. Sensitivity Analysis

Finally, the validity of the obtained MR results depended on a series of rigorous sensitivity analyses. Cochran’s *Q* test was used to detect heterogeneity among IVs (*p* > 0.05) [42]. MR‐Egger regression was used to assess for horizontal pleiotropy 43, and the MR‐PRESSO method identified potential outliers (SNPs with *p* < 0.05) [43]. Leave‐one‐out analyses were also performed to assess the influence of individual SNPs on the overall causal effect [44].

Reverse causality was assessed using Steiger’s directionality test, which evaluates whether the genetic instruments explain more variance in the exposure than in the outcome to confirm the correct causal direction. Potential outliers were then identified and removed in an iterative framework combining MR‐PRESSO, radial MR (applied to both IVW and MR‐Egger models to quantify each SNP’s contribution to heterogeneity via Cochran’s *Q* and Rücker’s Q), leave‐one‐out analysis, and Steiger filtering to exclude variants with evidence of reversed direction. Iterations continued until all of the following criteria were satisfied simultaneously: a nonsignificant MR‐PRESSO global test (*p* > 0.05), nonsignificant MR‐Egger heterogeneity (*Q* test, *p* > 0.05), and no evidence of directional pleiotropy according to the MR‐Egger intercept (*p* > 0.05) [45–47].

Given significant associations observed in the univariable MR analyses, multivariable MR (MVMR) was conducted to account for potential confounding by Type 2 diabetes, fasting insulin, and BMI (Supporting Table S1). MVMR exploits genetic variants associated with multiple correlated traits to estimate the direct effect of each exposure on the outcome while adjusting for the others, thereby enabling evaluation of independent effects and potential mediation pathways. For MVMR instrument selection, SNPs associated with GDM at *p* < 5 × 10^−7^ and with each confounder at *p* < 5 × 10^−8^ were initially extracted, restricted to variants with MAF > 0.01. LD clumping was performed separately for GDM and each confounder using an *R* ^2^ < 0.001 and a 10,000‐kb window, after which the clumped SNP sets were combined, deduplicated, and re‐clumped to construct a joint instrument set. Strand alignment procedures were applied by correcting nonpalindromic variants and excluding palindromic SNPs with ambiguous strand orientation, and only SNPs present across GDM and all confounder GWAS datasets were retained as final instruments for the MVMR analyses [48].

## 3. Results

### 3.1. IV Selection

Eight relevant IVs were selected for analysis of GDM as the exposure (the mean F was 68.48, the minimum F was 25.66, and the maximum F was 288.03). For the ER‐negative subtype outcome, no SNP was identified in the outcome summary data, nor could any proxy SNPs be found. For the ER‐positive subtype as the outcome, all SNPs were matched in the summary data. More data on the IVs screening process are shown in Supporting Table S2. Reverse causality was also examined using BCs as exposures. Eighteen IVs were selected for BC, 20 for ER‐negative subtype, and seven for ER‐positive subtype against GDM (Supporting Table S3).

### 3.2. Casual Effects of GDM on BC

As presented in Table 1, the genetic prediction results indicated the association between GDM and the overall incidence of BC (OR = 1.17, 95% CI = 1.03–1.32, *p* = 0.01) (Figure 2) and the incidence of the ER‐negative subtype (OR = 1.18, 95% CI = 1.04–1.35, *p* = 0.01) (Figure 3). In contrast, no such associations were found for the ER‐positive subtype (OR = 1.03, 95% CI = 0.95–1.10, *p* = 0.49) (Figure 4).

**TABLE 1:** Genetics predicts an association between GDM and breast cancer.

Exposure	Outcome	Number of SNPs	Methods	OR (95% CI)	*p*
GDM	Breast cancer overall	7	IVW	1.17 (1.03–1.32)	0.01
			MR‐Egger	0.98 (0.77–1.26)	0.89
			Weighted median	1.06 (0.93–1.21)	0.40
			Weighted mode	1.03 (0.91–1.17)	0.67

GDM	ER‐ breast cancer	7	IVW	1.18 (1.04–1.35)	0.01
			MR‐Egger	1.11 (0.81–1.52)	0.55
			Weighted median	1.16 (1.04–1.29)	0.01
			Weighted mode	1.13 (1.01–1.26)	0.08

GDM	ER+ breast cancer	8	IVW	1.03 (0.95–1.1)	0.49
			MR‐Egger	0.99 (0.82–1.19)	0.90
			Weighted median	1.02 (0.97–1.07)	0.44
			Weighted mode	1.03 (0.97–1.08)	0.38

ER‐ breast cancer	GDM	17	IVW	0.96 (0.92–1.01)	0.15
			MR‐Egger	0.99 (0.86–1.13)	0.84
			Weighted median	0.99 (0.92–1.06)	0.78
			Weighted mode	1.02 (0.90–1.14)	0.79

Breast cancer overall	GDM	16	IVW	1.00 (0.95–1.05)	0.87
			MR‐Egger	0.97 (0.84–1.10)	0.61
			Weighted median	1.00 (0.94–1.05)	0.91
			Weighted mode	0.96 (0.89–1.04)	0.33

ER+ breast cancer	GDM	98	IVW	0.96 (0.92–1.00)	0.07
			MR‐Egger	0.97 (0.88–1.06)	0.49
			Weighted median	0.93 (0.88–0.98)	0.01
			Weighted mode	0.91 (0.85–0.99)	0.02

Abbreviations: GDM, gestational diabetes mellitus; IVW, inverse‐variance weighted; SNPs, single‐nucleotide polymorphisms.

FIGURE 2The causal effect of GDM on breast cancer overall. (a) Forest plot; (b) leave‐one‐out sensitivity analysis; (c) scatter plot; (d) funnel plot.

**Figure:**
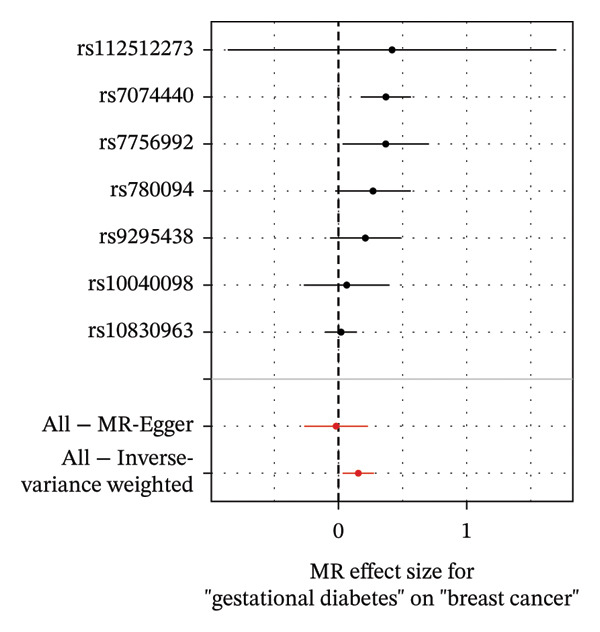
(a)

**Figure:**
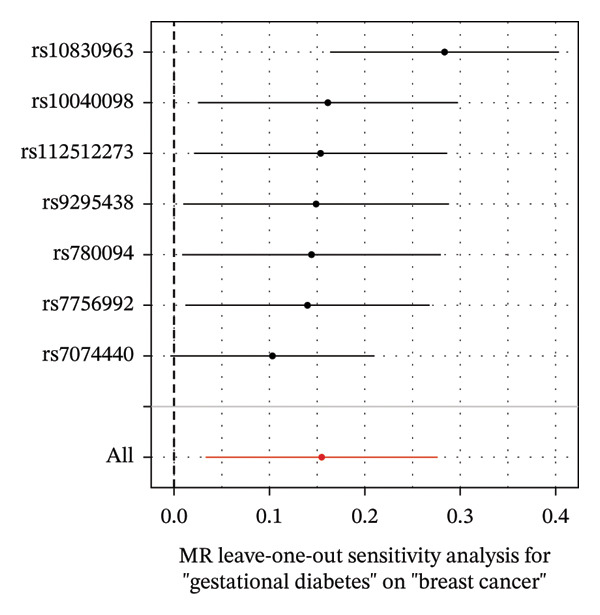
(b)

**Figure:**
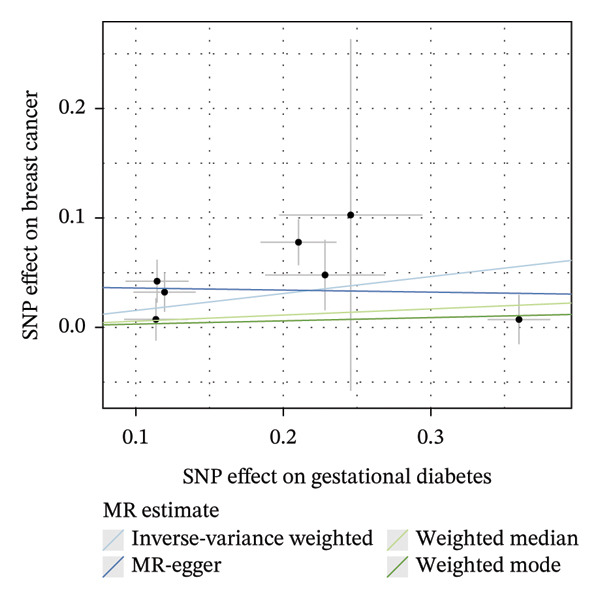
(c)

**Figure:**
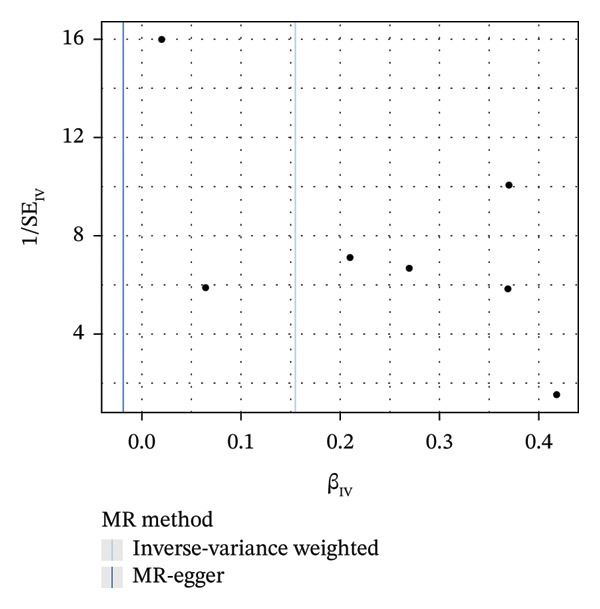
(d)

FIGURE 3The causal effect of GDM on ER‐negative breast cancer. (a) Forest plot; (b) leave‐one‐out sensitivity analysis; (c) scatter plot; (d) funnel plot.

**Figure:**
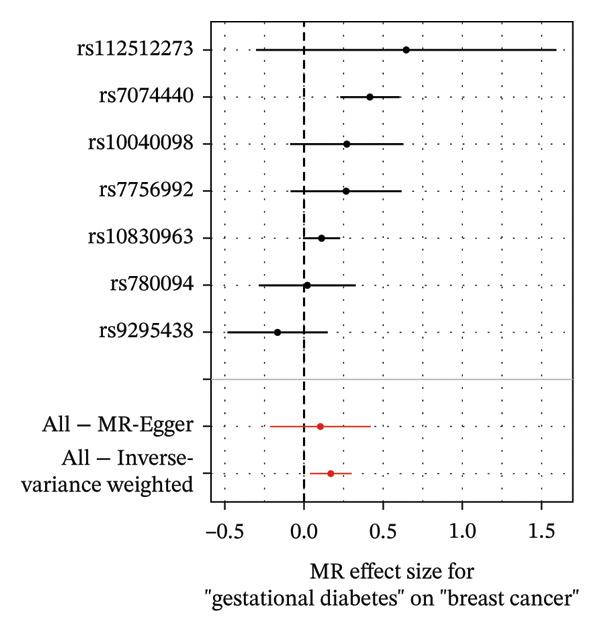
(a)

**Figure:**
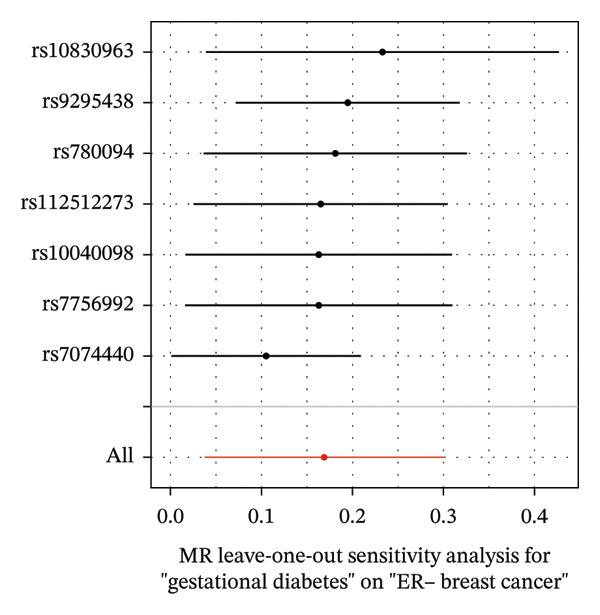
(b)

**Figure:**
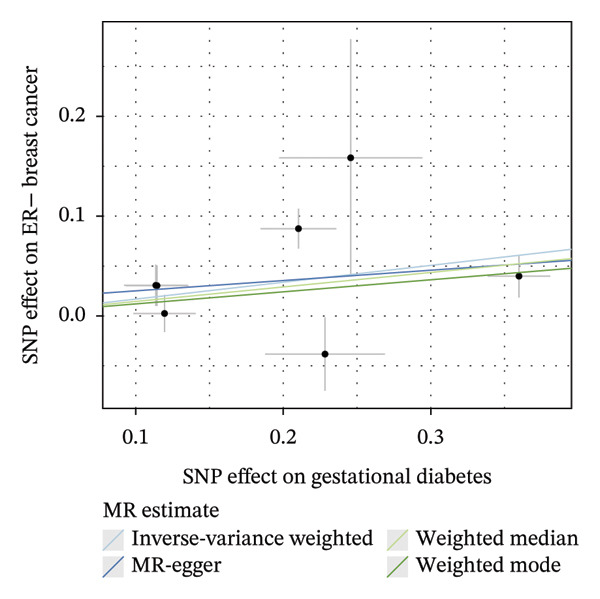
(c)

**Figure:**
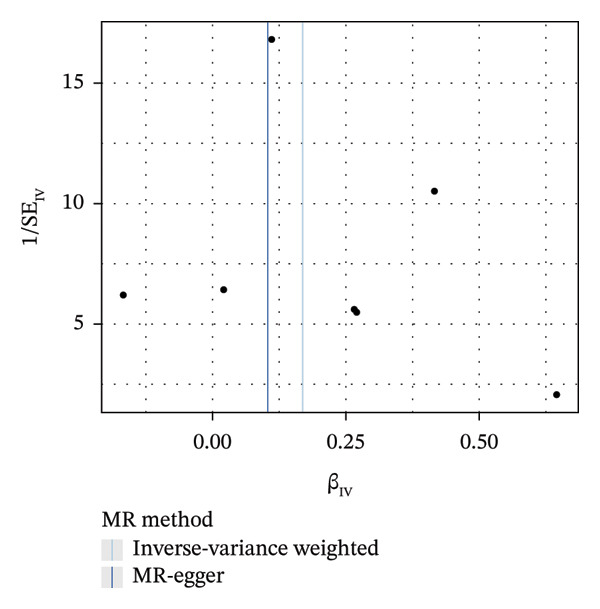
(d)

FIGURE 4The causal effect of GDM on ER‐positive breast cancer. (a) Forest plot; (b) leave‐one‐out sensitivity analysis; (c) scatter plot; (d) funnel plot.

**Figure:**
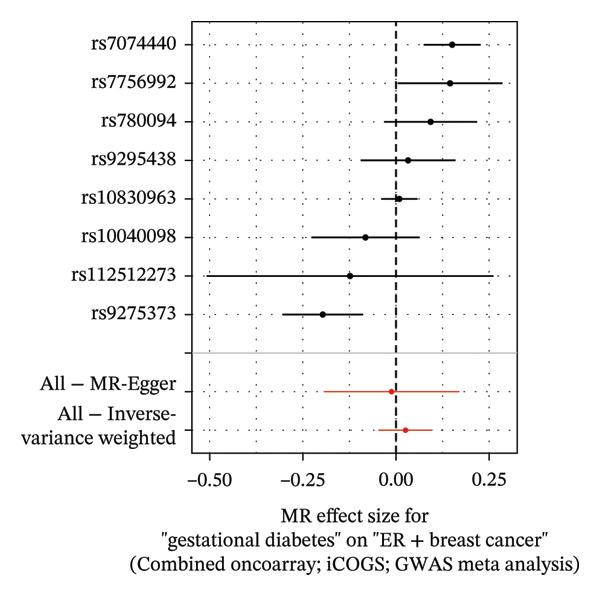
(a)

**Figure:**
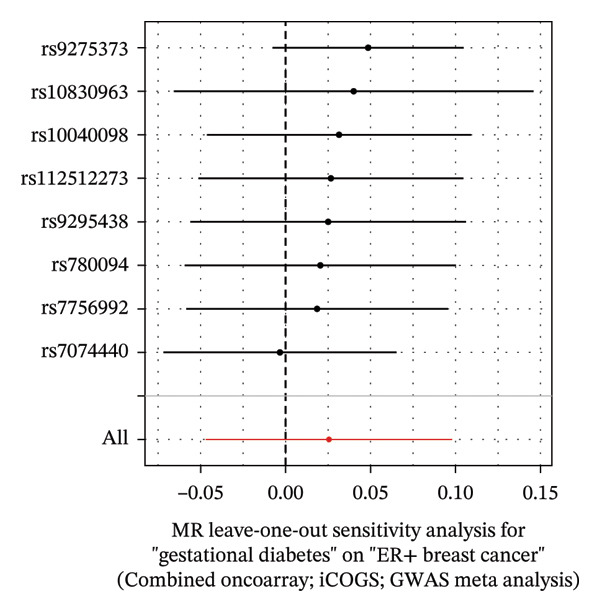
(b)

**Figure:**
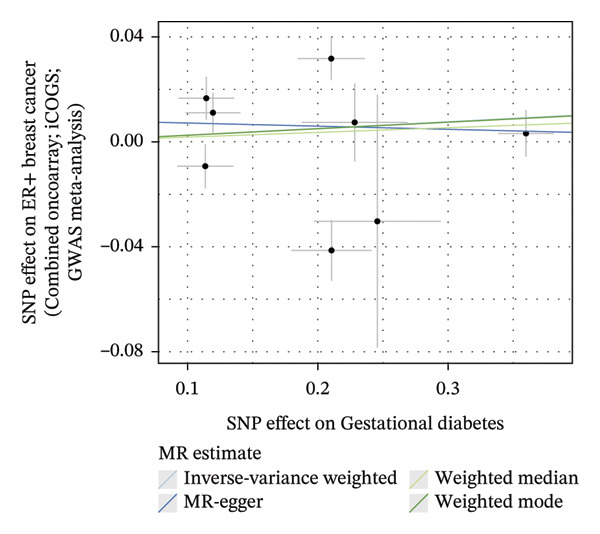
(c)

**Figure:**
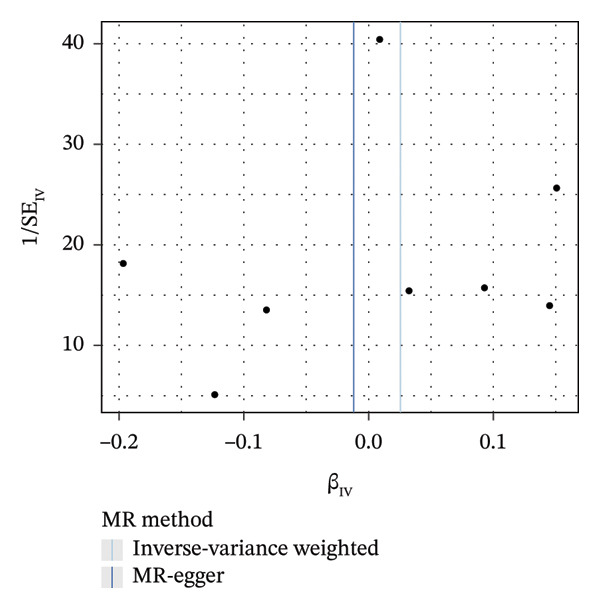
(d)

To further assess the reliability of these findings, sensitivity analyses were performed. As shown in Table 2, heterogeneity was identified in the association between GDM and both the ER‐negative (*p* = 0.02) and ER‐positive (*p* < 0.001) subtypes. Furthermore, the MR‐PRESSO results detected two outliers in the association between GDM and the ER‐positive subtype (*p* = 0.003) (Table 3). No significant horizontal pleiotropy was identified. The results were visually supported by the leave‐one‐out and funnel plots (Figures 2, 4(b), 4(d)). All associations showed correct causal direction (*p* < 1 × 10^−20^), supporting reliability (Table 4). Although MR‐PRESSO did not detect outliers for ER‐negative BC (Global *p* = 0.12), Cochran’s *Q* test indicated significant heterogeneity (*p* = 0.02, Table 2). Therefore, we employed radial MR as a more sensitive method to detect and remove outliers. The application of the radial MR analysis and outlier removal did not change the conclusions of the original forward analyses (Supporting Tables S4–S6). In the MVMR, the total effect of genetically predicted GDM on overall BC risk remained statistically significant after adjustment for fasting insulin or BMI in MVMR, whereas the association was attenuated to nonsignificance after additional adjustment for Type 2 diabetes. For ER‐negative BC, the GDM‐associated risk persisted after controlling for BMI. Still, it became nonsignificant when fasting insulin or Type 2 diabetes were included as covariates, suggesting that insulin‐related pathways and subsequent diabetes liability may partly mediate the GDM–ER‐negative BC relationship. In contrast, BMI alone does not fully account for the observed association (Table 5).

**TABLE 2:** The heterogeneity and pleiotropy results.

Exposure	Outcome	Heterogeneity		Pleiotropy	
		Q statistic (IVW)	*p*	MR‐Egger intercept	*p*
GDM	Breast cancer overall	12.079	0.06	0.038	0.19
GDM	ER‐ breast cancer	14.535	0.02	0.015	0.67
GDM	ER+ breast cancer	33.624	< 0.001	0.008	0.67
ER‐ breast cancer	GDM	14.647	0.55	−0.003	0.75
Breast cancer overall	GDM	24.478	0.06	0.006	0.63
ER+ breast cancer	GDM	183.965	< 0.001	−0.001	0.87

Abbreviations: GDM, gestational diabetes mellitus; IVW, inverse‐variance weighted.

**TABLE 3:** The MR pleiotropy residual sum and outlier results—association between GDM (risk factor) and breast cancer (outcome).

Exposure	Outcome	Breast cancer or GDM		Outlier corrected		Global *p*	Number of outliers	Distortion *p*
		OR (CI%)	*p*	OR (CI%)	*p*			
GDM	Breast cancer overall	1.17 (1.03–1.32)	0.05	NA	NA	0.15	NA	NA
GDM	ER‐ breast cancer	1.18 (1.04–1.35)	0.05	NA	NA	0.12	NA	NA
GDM	ER+ breast cancer	1.03 (0.95–1.1)	0.51	1.02 (0.98–1.07)	0.40	0.003	2	0.78
Breast cancer overall	GDM	0.99 (0.95–1.04)	0.82	0.99 (0.95–1.04)	0.82	0.09	NA	NA
ER‐ breast cancer	GDM	0.99 (0.91–1.08)	0.87	0.993 (0.91–1.08)	0.87	< 0.001	1	0.60
ER+ breast cancer	GDM	0.97 (0.93–1.01)	0.16	0.97 (0.93–1.01)	0.16	< 0.001	1	0.64

Abbreviations: GDM, gestational diabetes mellitus; IVW, inverse‐variance weighted.

**TABLE 4:** Steiger directionality test results (forward).

Exposure	Outcome	Correct causal direction	Steiger *p*‐value
Gestational diabetes	Breast cancer	TRUE	4.4 × 10^−38^
	ER+ breast cancer	TRUE	2.85 × 10^−45^
	ER‐ breast cancer	TRUE	2.53 × 10^−22^

**TABLE 5:** MVMR results.

Exposure	Outcome	Adjusted for	OR (95% CI)	*p*‐value
Gestational diabetes	Breast cancer	T2D	1.018 (0.931–1.114)	0.6877
		Fasting insulin	1.064 (1.001–1.132)	0.0473
		BMI	1.091 (1.041–1.142)	0.0002

Gestational diabetes	ER‐negative breast cancer	T2D	1.040 (0.937–1.153)	0.4648
		Fasting insulin	1.075 (0.995–1.161)	0.0671
		BMI	1.102 (1.034–1.174)	0.0029

### 3.3. Casual Effects of BC on GDM

In the reverse MR analysis, however, there was no evidence of causality between BC and GDM (Table 1). However, Cochran’s *Q* test revealed heterogeneity between the ER‐positive subtype and GDM (*p* < 0.001); no horizontal pleiotropy was detected (Table 2). One outlier was observed for both the ER‐negative and ER‐positive subtypes (both *p* < 0.001); however, the associations remained nonsignificant after the removal of these outliers (Table 3). The corresponding visual analyses for these results are provided in Supporting Figures S1–S3. All associations showed correct causal direction (*p* < 1 × 10^−47^), supporting validity (Supporting Table S7). The result conclusions remained unchanged after radial MR analysis (Supporting Tables S8–S10).

## 4. Discussion

Our study assessed the bidirectional contributory association between GDM and BC. The results suggested that GDM may influence BC development, particularly ER‐negative BC. The results suggest that a history of GDM might be a valuable factor to incorporate into personalized BC risk assessment models.

For the causal association between GDM and ER‐negative BC, these results contradict the observational study by Park et al. [14], which reported an association between GDM and BC, but mainly driven by ER‐positive BC and, to a lesser extent, by ER‐negative BC. On the other hand, Wang et al. [9] conducted a meta‐analysis and reported no association between GDM and BC, contradicting the present study. Nevertheless, although the risk of BC in women who had GDM during pregnancy has been discussed in several observational studies [9–12, 49], these studies were affected by the numerous confounders, including postpartum metabolic health, making it difficult to draw consistent and reliable conclusions [5, 13, 50]. Mechanistically, the association between GDM and BC is supported by the known relationship between diabetes and cancers [51–53]. Indeed, diabetes, including GDM, is associated with hyperinsulinemia and chronic inflammation, which promote the proliferation of cancer cells [54–56]. Furthermore, there were reports that insulin promotes proliferation of ER‐negative BC cells via the PI3K/AKT/mTOR pathway, whereas ER‐positive tumors, which depend on estrogen signaling, exhibit lower sensitivity to insulin‐driven growth signals [57]. Inflammatory factors associated with GDM, including IL‐6 and TNF‐α, may enhance ER‐negative tumor invasiveness through the NF–κB pathway [58, 59]. Nevertheless, further experimental validation is needed to confirm the dominant role of this mechanism in ER‐negative tumor progression.

Furthermore, the reverse MR analysis did not indicate that BC was causally associated with GDM risk. Pregnancy usually occurs in women in their 20 s and 30 s, while the risk of BC becomes significant in the 50 s [5]. Thus, the causal association between GDM and BC is more plausible, aligning with the natural history of the disease and the temporal sequence of pregnancy and BC onset. Nevertheless, a few women will suffer from BC at a young age and before a pregnancy. ER‐positive/progesterone receptor (PR)–positive BC subtypes are driven by estrogen and progesterone levels [60], which also affect glucose metabolism and insulin sensitivity and, in turn, may potentially lead to GDM [61]. In addition, cancer treatments may contribute to a higher risk of GDM in pregnant women with a history of BC [62]. A study concluded that BC itself is unlikely to directly cause GDM [63]. This finding reinforces the idea that GDM‐related metabolic disturbances, such as hyperinsulinemia and chronic inflammation, may promote ER‐negative tumor progression, rather than BC influencing glucose metabolism or insulin resistance that would lead to GDM.

Even though GDM is a metabolic disease, it stands apart from the other types of diabetes since it is not only due to insulin resistance and hyperglycemia but involves complex interplays with the female hormones during pregnancy and pregnancy‐specific metabolism [61, 64, 65], with the female hormones during pregnancy also playing an independent role in the BC risk [66, 67]. Therefore, even though several studies on the association between diabetes and BC risk are available [68–71], it was important to confirm the causal association with GDM, especially since previous MR studies report conflicting results, some reporting causal associations between diabetes (or markers) and BC [72–74] and others not [75, 76]. In addition, most previous MR studies examined overall BC without distinguishing by hormone receptor status. Of note, the present study showed that GDM increased the risk of ER‐negative BC, not ER‐positive BC, whereas previous observational studies showed that Types 1 and 2 diabetes increased the risk of BC 68–71, which might reflect the influence of the specific hormonal pattern during pregnancy. Although the present study reports causal associations, they will have to be investigated mechanistically in future studies.

The present study is more hypothesis‐generating than definitive, and its data cannot be used to determine the biological mechanisms involved. Nevertheless, based on the literature, several interconnected metabolic and pregnancy‐related pathways could plausibly link GDM to a higher risk of ER‐negative BC, although direct mechanistic evidence remains limited and partly speculative [14, 77]. Women with GDM experience exaggerated insulin resistance and compensatory hyperinsulinemia during pregnancy, driven by placental hormones and pre‐existing metabolic susceptibility. Elevated insulin and insulin‐like growth factor (IGF) signaling can promote proliferation, survival, and metastatic behavior of BC cells via PI3K–Akt–mTOR and related pathways, and hyperinsulinemic states and high insulinemic diets have been particularly linked to increased risk of aggressive or ER‐negative BC [77–79]. GDM often coexists with obesity, central adiposity, and a chronic low‐grade inflammatory state characterized by elevated proinflammatory cytokines and altered adipokine profiles. Inflammatory signaling, including NF‐κB and related pathways, can foster a protumorigenic microenvironment and has been implicated in the development and progression of more aggressive BC subtypes, including ER‐negative BC [77, 80, 81]. Sustained or recurrent hyperglycemia in GDM can increase oxidative stress and the formation of advanced glycation end products, which can damage DNA and impair genomic stability. In women carrying high‐risk germline variants (e.g., BRCA1/2), such metabolic stressors may further compromise DNA repair capacity through insulin/IGF‐mediated PI3K–Akt signaling, potentially favoring ER‐negative or triple‐negative phenotypes that are common in pregnancy‐associated and metabolically driven BCs [77, 82, 83]. Pregnancy and lactation drive extensive remodeling of the mammary gland, with waves of proliferation, differentiation, and involution that are modulated by estrogens, progesterone, prolactin, and growth factors. In the context of GDM, the combination of rapid hormonal shifts and an altered metabolic–inflammatory milieu may create a “wound‐healing‐like” environment during postpartum involution that promotes survival and expansion of transformed or premalignant cells, a process that has been particularly associated with ER‐negative and triple‐negative tumors after pregnancy [82–84]. Overall, current evidence supports a biologically plausible link through insulin resistance, hyperinsulinemia, inflammation, oxidative stress, and pregnancy‐related mammary remodeling, but these mechanisms remain incompletely defined and require experimental validation specifically in the context of GDM and ER‐negative BC. Importantly, in the present study, the MVMR analysis showed that the association between GDM and BC disappears (i.e., *p* > 0.05) after adjusting for insulin, directly supporting the hypothesis that the insulin pathway acts as a mediating mechanism in the associations observed here.

Although the IVW method indicated a statistically significant positive association, the MR‐Egger and weighted median estimators did not provide concordant evidence, suggesting that the overall findings should be interpreted with caution, given their sensitivity to the MR method applied. Still, in MR, IVW is typically treated as the primary analysis because, when most or all instruments are valid, and there is no substantial directional pleiotropy, it provides an unbiased estimate with maximal statistical power and the narrowest CIs. Under these conditions, MR‐Egger and the weighted median are less efficient, so they are mainly used as complementary sensitivity analyses rather than replacements for IVW. Moreover, MR‐Egger relies on the strong, often untestable InSIDE assumption and has low power, leading to wide CIs and unstable estimates, whereas the weighted median trades some bias robustness for reduced efficiency and can perform worse than IVW if the majority of instruments are valid. Consequently, if there is no strong evidence of pleiotropy (e.g., null Egger intercept, low heterogeneity after outlier removal), it is reasonable to place more weight on the IVW estimate and view MR‐Egger and weighted median results as checks on robustness rather than as the primary basis for inference [27, 28, 39, 45].

The wide CIs and their proximity to the null indeed indicate that the precision of the causal estimates is limited, and the possibility of a weak or even null true effect cannot be excluded. Hence, these results should be interpreted cautiously and are more hypothesis‐generating than definitive. At the same time, the point estimates are directionally consistent across several MR methods and sensitivity analyses, and there is no strong evidence of pleiotropy or violation of core MR assumptions, which provides some support for the idea that the observed association may reflect an underlying causal signal rather than pure noise. Nonetheless, the limited power and imprecision are acknowledged as key constraints of this study and highlight the need for larger, well‐powered MR analyses and complementary observational or experimental studies to confirm or refute this potential causal relationship.

This study has several limitations. First, it was constrained by the scarcity of independent SNPs associated with certain exposure factors, resulting in low statistical power or weak statistical significance. The available GWAS data remain limited, highlighting the need for large‐scale datasets. Furthermore, the *P*‐value threshold for SNP selection had to be relaxed to obtain enough SNPs for MR analysis. Despite the risk of introducing weak IVs, all F‐values remained > 10, indicating the absence of weak instrumental bias. Second, the generalizability of the findings is limited, as the study population was drawn from a single Northern European dataset; hence, additional research is warranted to determine whether these findings generalize to other ethnicities, potentially requiring further MR analyses to corroborate them. GDM may influence BC risk through several biological mechanisms, such as chronic inflammation, hormonal changes, and obesity, which need to be taken into consideration. Future research should replicate the findings in multiethnic cohorts and integrate metabolomics data to elucidate the impact of GDM‐related metabolites (including free fatty acids and adiponectin) on BC subtypes. Finally, Cochran’s *Q* confirmed heterogeneity in the GDM vs. ER‐negative BC analysis (*p* = 0.024), suggesting that some variants may be violating MR assumptions.

In conclusion, GDM may influence BC development, particularly ER‐negative BC. This study suggests that women with GDM could benefit from closer BC surveillance and personalized approaches.

## Author Contributions

Wangwang Peng performed the statistical analysis, participated in study design, and drafted the manuscript. Zhulan Huang participated in the acquisition, analysis, and interpretation of data. Min Zhang revised the paper.

## Funding

The authors have nothing to report.

## Disclosure

All authors read and approved the final manuscript.

## Ethics Statement

The authors have nothing to report.

## Consent

The authors have nothing to report.

## Conflicts of Interest

The authors declare no conflicts of interest.

## Supporting Information

Supporting materials are provided to support the main findings of this study. Supporting Figures S1–S3 present additional Mendelian randomization analyses, including forest plots, leave‐one‐out sensitivity analyses, scatter plots, and funnel plots. Supporting Tables S1–S10 provide detailed information on the GWAS datasets, characteristics of the genetic instrumental variables, causal estimates after outlier removal, and results of heterogeneity, pleiotropy, MR‐PRESSO, and Steiger directionality tests.

Supporting Figure Legends.

Figure S1. The causal effect of breast cancer overall on GDM. (A) Forest plot; (B) leave‐one‐out sensitivity analysis; (C) scatter plot; (D) funnel plot.

Figure S2. The causal effect of ER‐negative breast cancer on GDM. (A) Forest plot; (B) leave‐one‐out sensitivity analysis; (C) scatter plot; (D) funnel plot.

Figure S3. The causal effect of ER‐positive breast cancer on GDM. (A) Forest plot; (B) leave‐one‐out sensitivity analysis; (C) scatter plot; (D) funnel plot.

Supporting Table Legends.

Table S1. Detailed characteristics of the GWAS datasets used in the Mendelian randomization analysis.

Table S2. Characteristics of the genetic instrumental variables associated with gestational diabetes mellitus (exposure) used in the forward Mendelian randomization analysis.

Table S3. Characteristics of the genetic instrumental variables associated with breast cancer and its subtypes (exposures) used in the reverse Mendelian randomization analysis.

Table S4. Causal estimates of the effect of gestational diabetes mellitus on breast cancer risks derived from Mendelian randomization analysis after outlier removal.

Table S5. Heterogeneity and horizontal pleiotropy tests for the association between gestational diabetes mellitus and breast cancer subtypes after outlier removal.

Table S6. MR‐PRESSO global test results and outlier detection for the forward Mendelian randomization analysis.

Table S7. Results of the Steiger directionality test verifying the causal direction in the reverse Mendelian randomization analysis.

Table S8. Causal estimates of the effect of breast cancer subtypes on gestational diabetes mellitus risk derived from the reverse Mendelian randomization analysis after outlier removal.

Table S9. Heterogeneity and horizontal pleiotropy tests for the reverse Mendelian randomization analysis after outlier removal.

Table S10. MR‐PRESSO global test results for the reverse Mendelian randomization analysis after outlier removal.

## Supporting information


**Supporting Information** Additional supporting information can be found online in the Supporting Information section.

## Data Availability

All data generated or analyzed during this study are included in this published article.
