# Supplementary figures and images for: Genetic Evidence Links Gestational Diabetes Mellitus to Increased ER‐Negative Breast Cancer Risk Through a Mendelian Randomization Analysis
Source: Int J Endocrinol. 2026 Apr 28;2026:7371037. doi: 10.1155/ije/7371037 (PMC13122568; doi:10.1155/ije/7371037)

# MR Estimate

- Inverse variance weighted
- MR Egger
- Weighted median
- Weighted mode

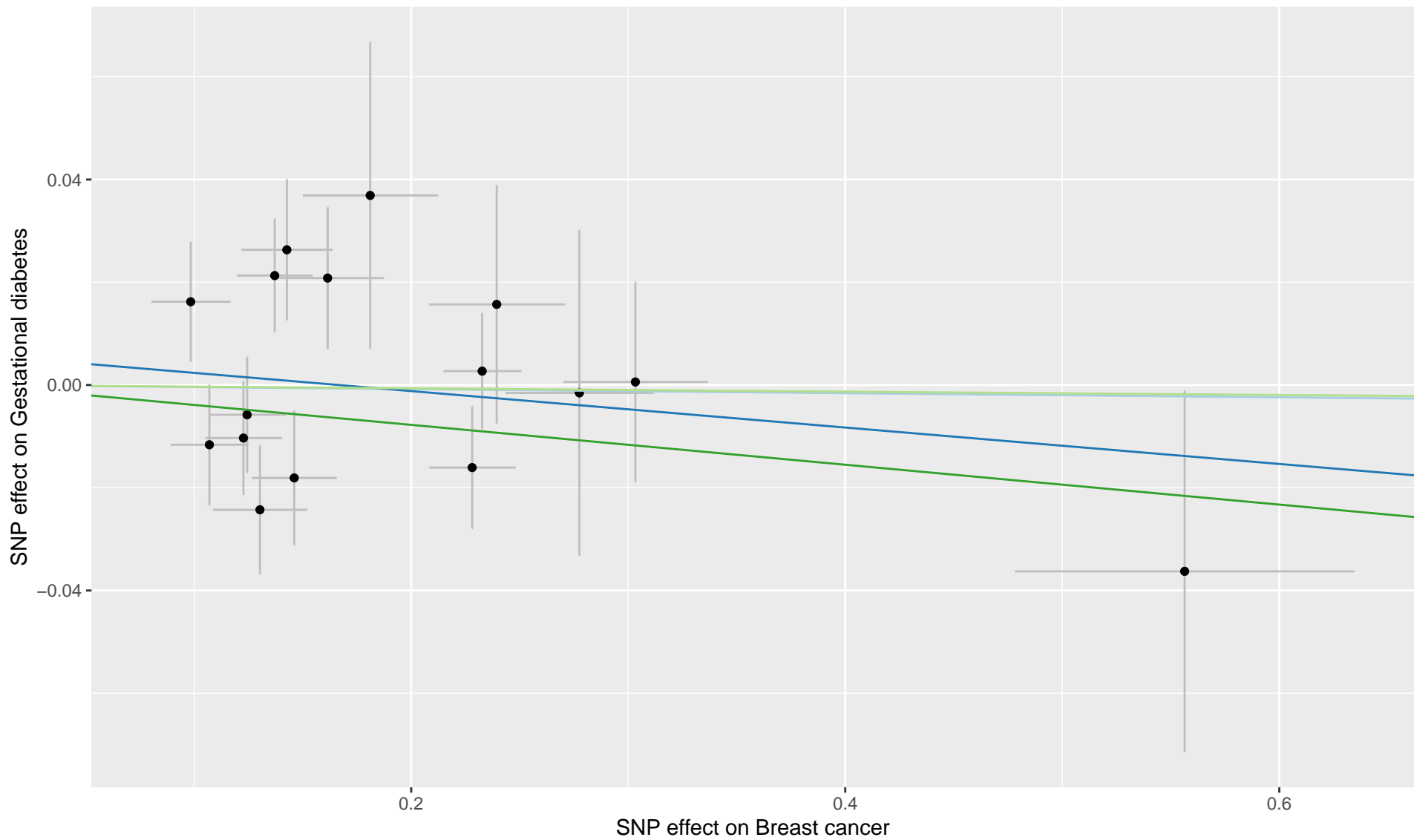

Supplement: Supplementary file 1 — Supporting Information Additional supporting information can be found online in the Supporting Information section. [file IJE-2026-7371037-s001.zip › Figuer S1C.pdf]

# MR Estimate

- Inverse variance weighted
- MR Egger
- Weighted median
- Weighted mode

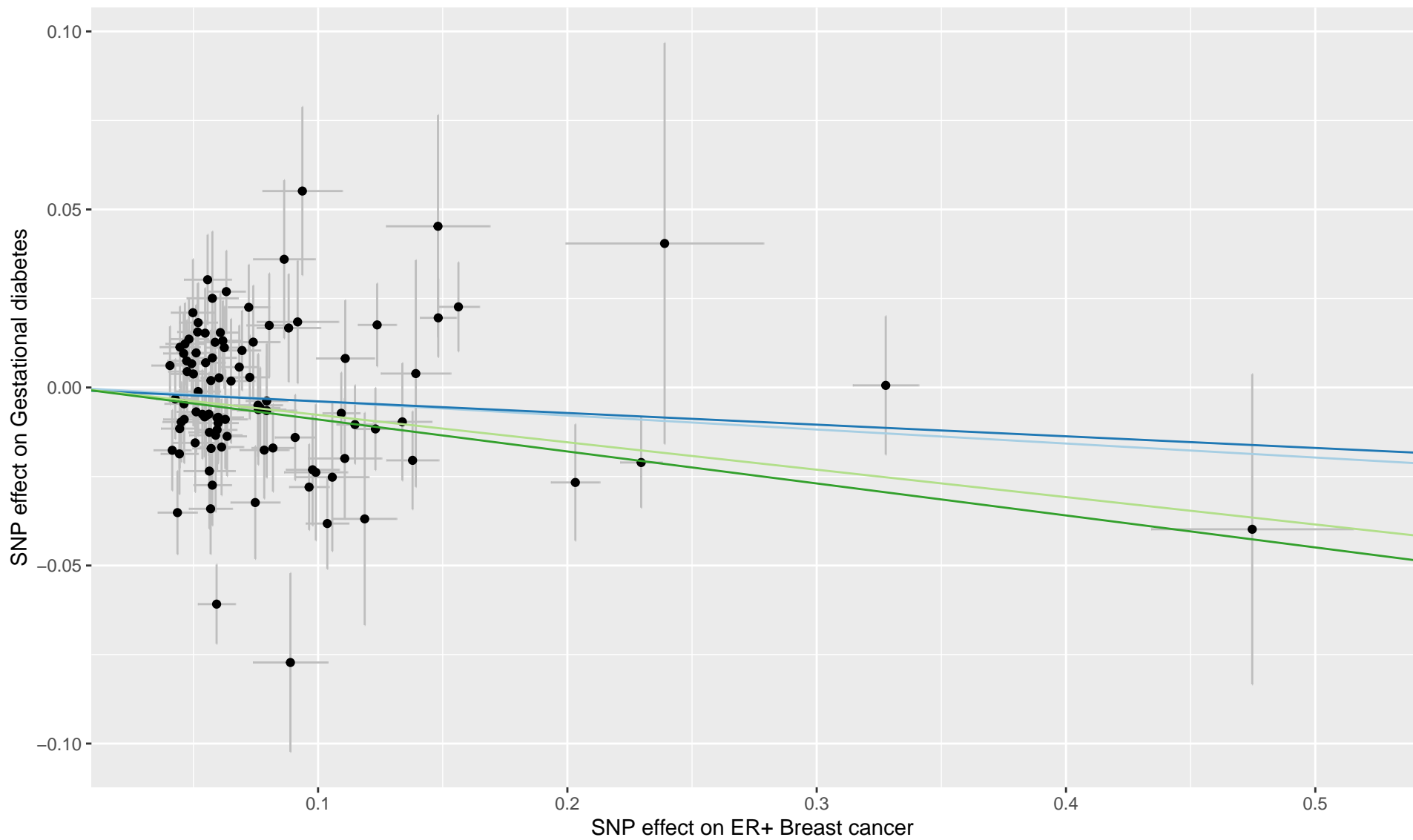

Supplement: Supplementary file 1 — Supporting Information Additional supporting information can be found online in the Supporting Information section. [file IJE-2026-7371037-s001.zip › Figuer S3C.pdf]

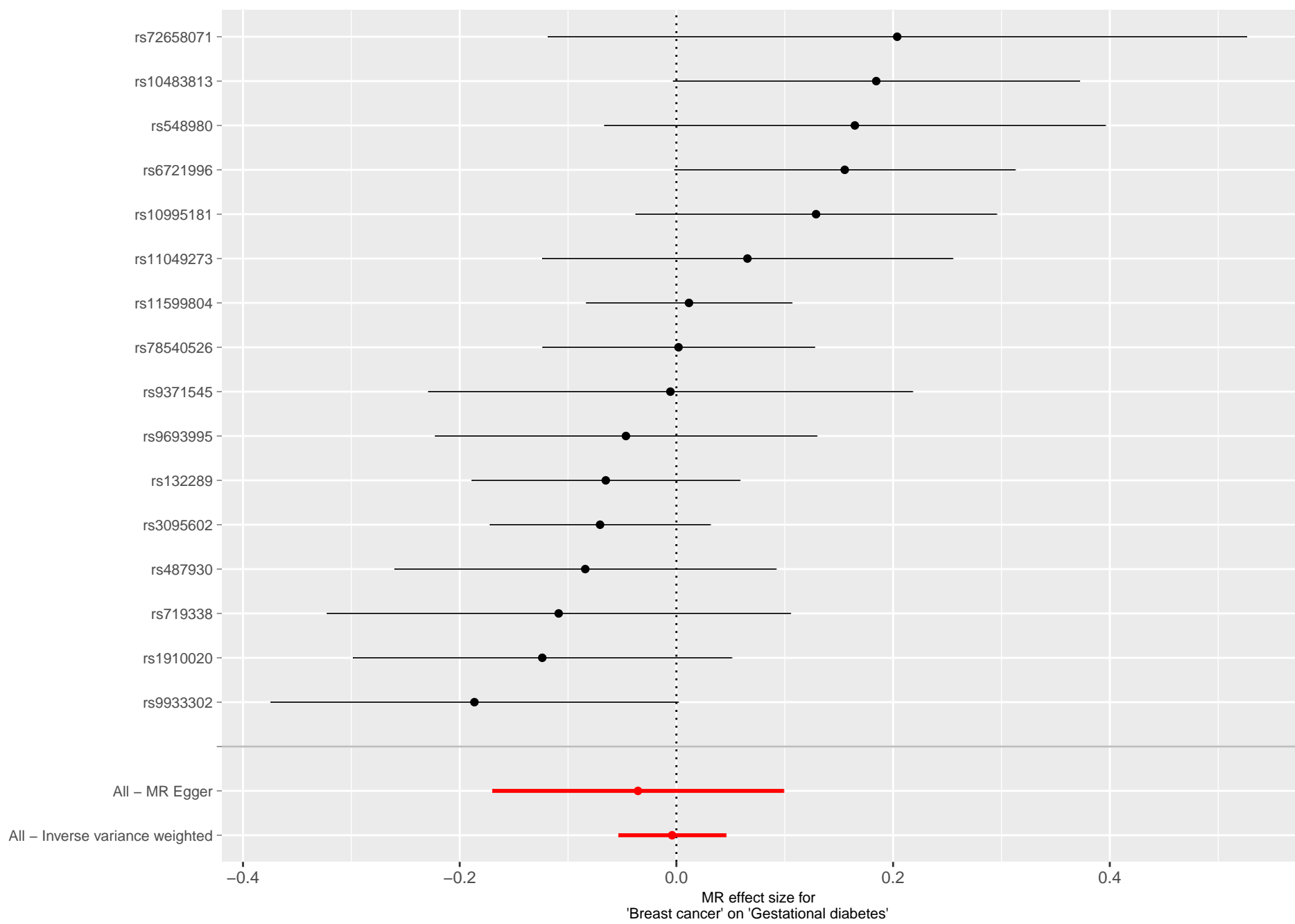

Supplement: Supplementary file 1 — Supporting Information Additional supporting information can be found online in the Supporting Information section. [file IJE-2026-7371037-s001.zip › Figure S1A.pdf]

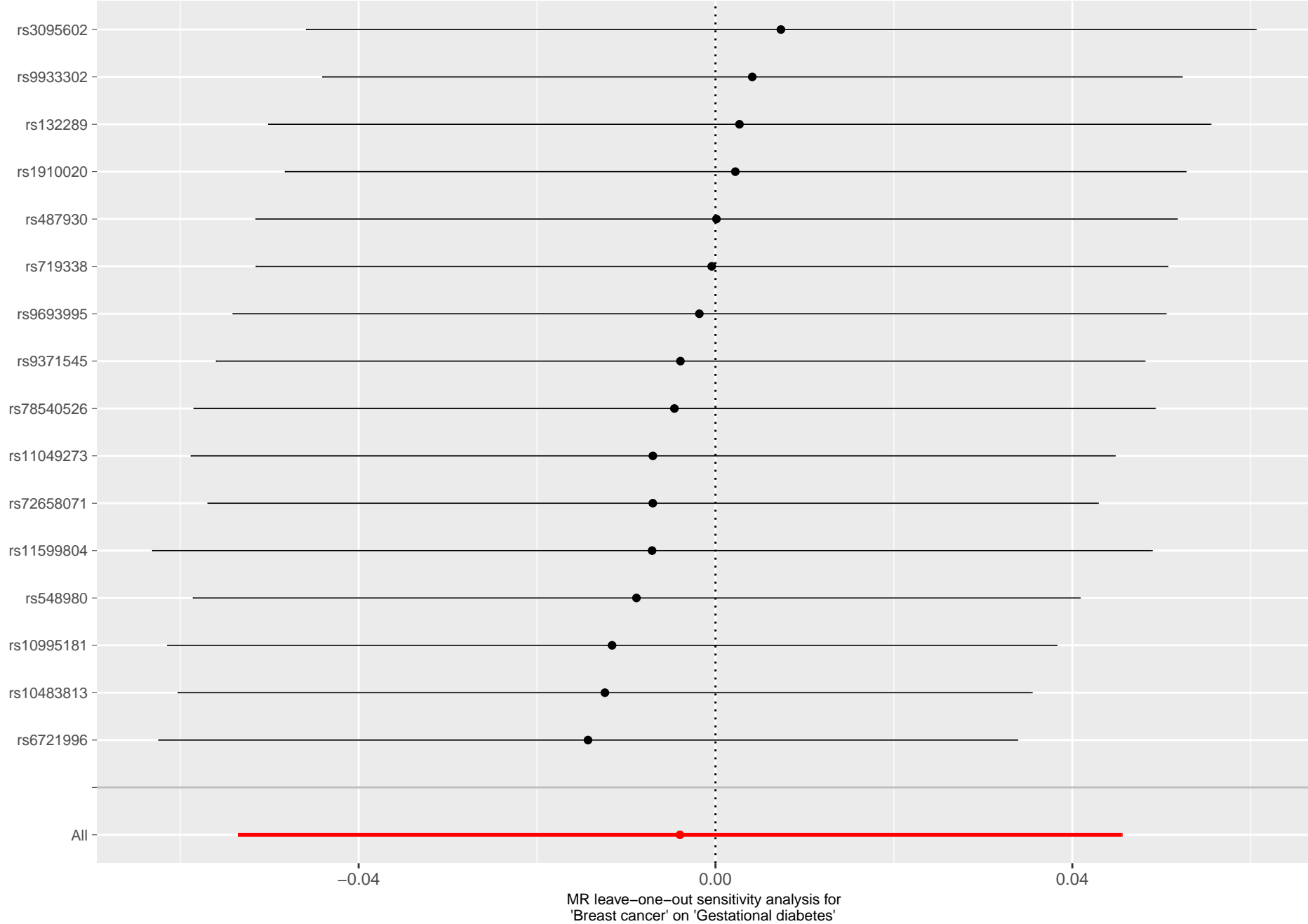

Supplement: Supplementary file 1 — Supporting Information Additional supporting information can be found online in the Supporting Information section. [file IJE-2026-7371037-s001.zip › Figure S1B.pdf]

# MR Method

- Inverse variance weighted
- MR Egger

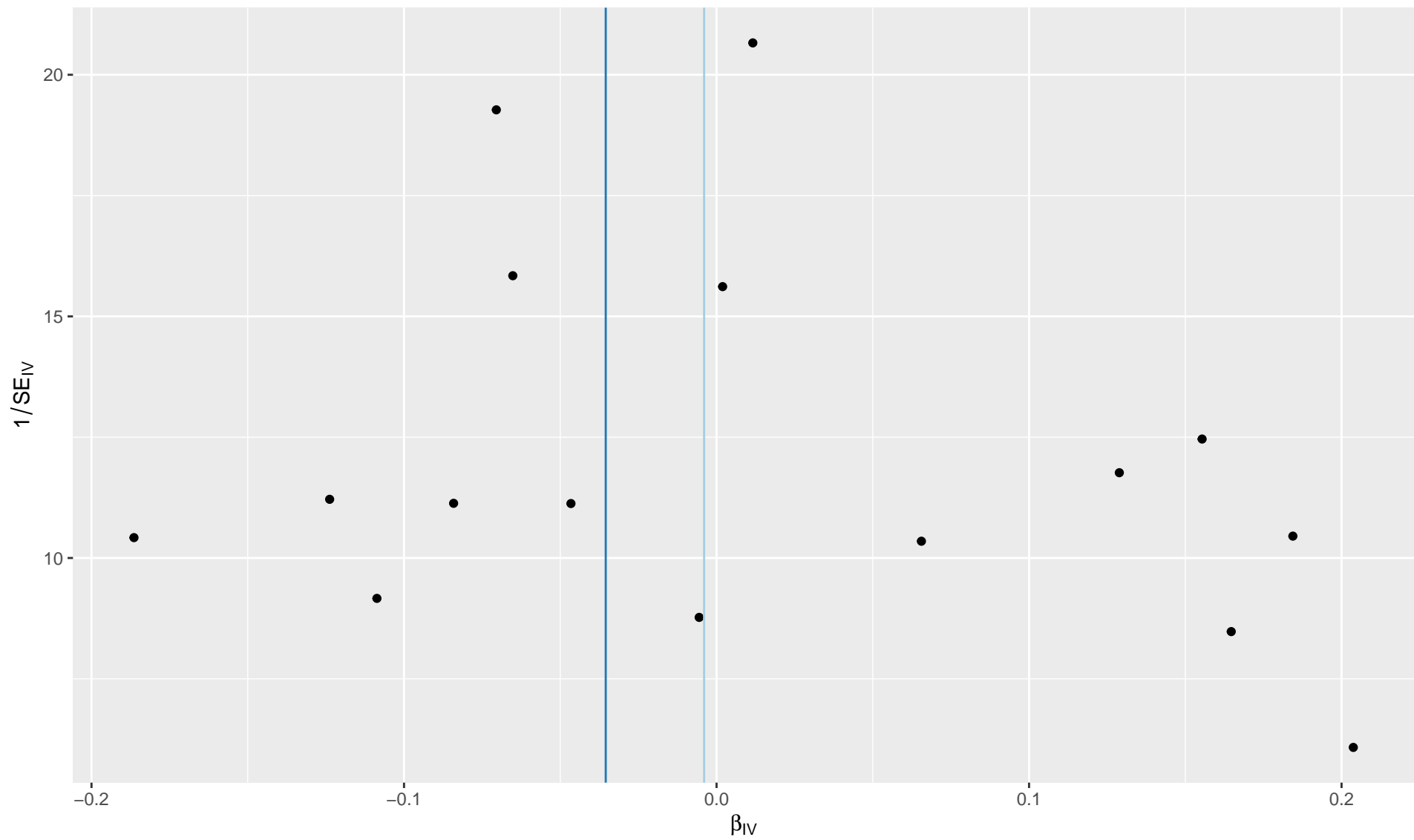

Supplement: Supplementary file 1 — Supporting Information Additional supporting information can be found online in the Supporting Information section. [file IJE-2026-7371037-s001.zip › Figure S1D.pdf]

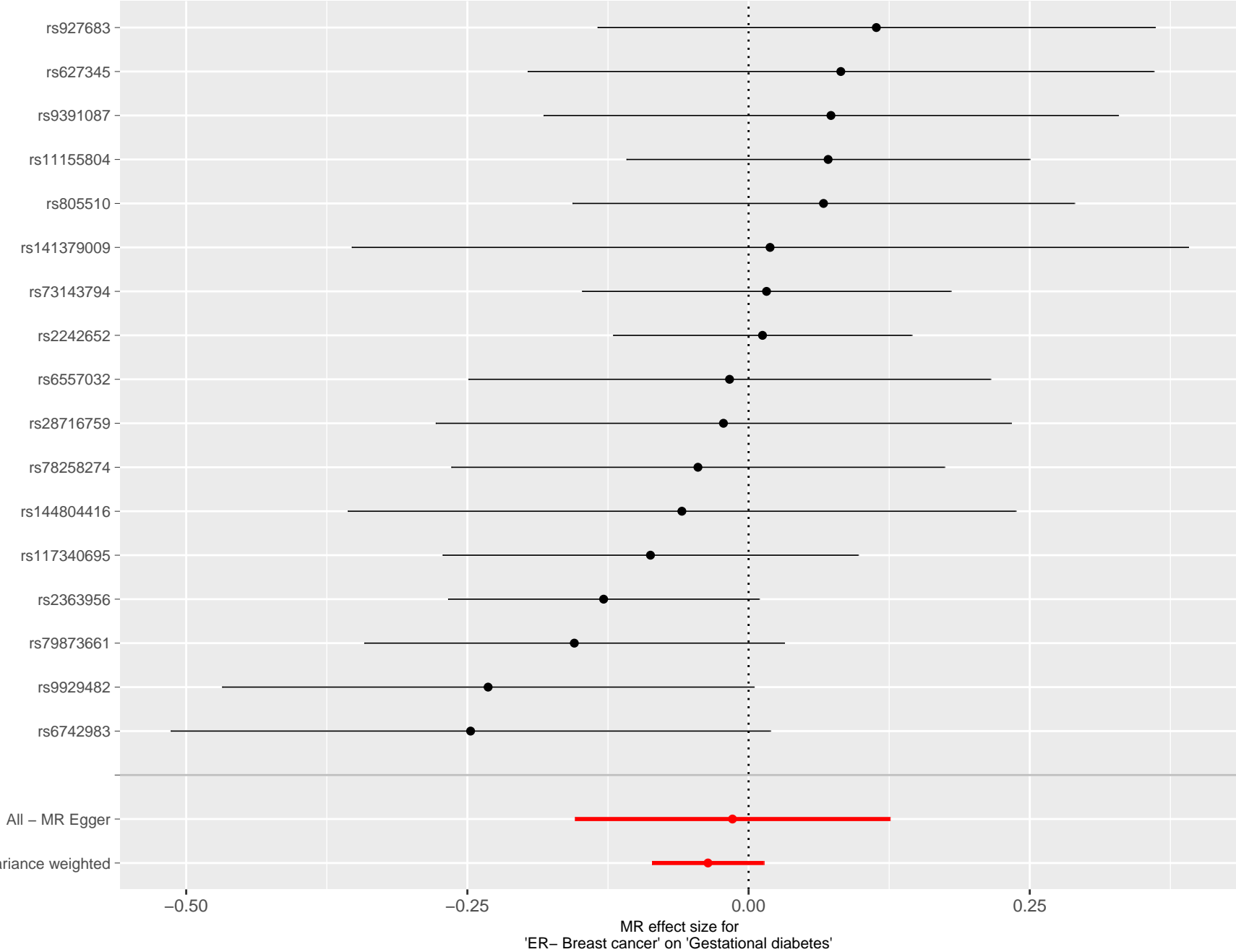

Supplement: Supplementary file 1 — Supporting Information Additional supporting information can be found online in the Supporting Information section. [file IJE-2026-7371037-s001.zip › Figure S2A.pdf]

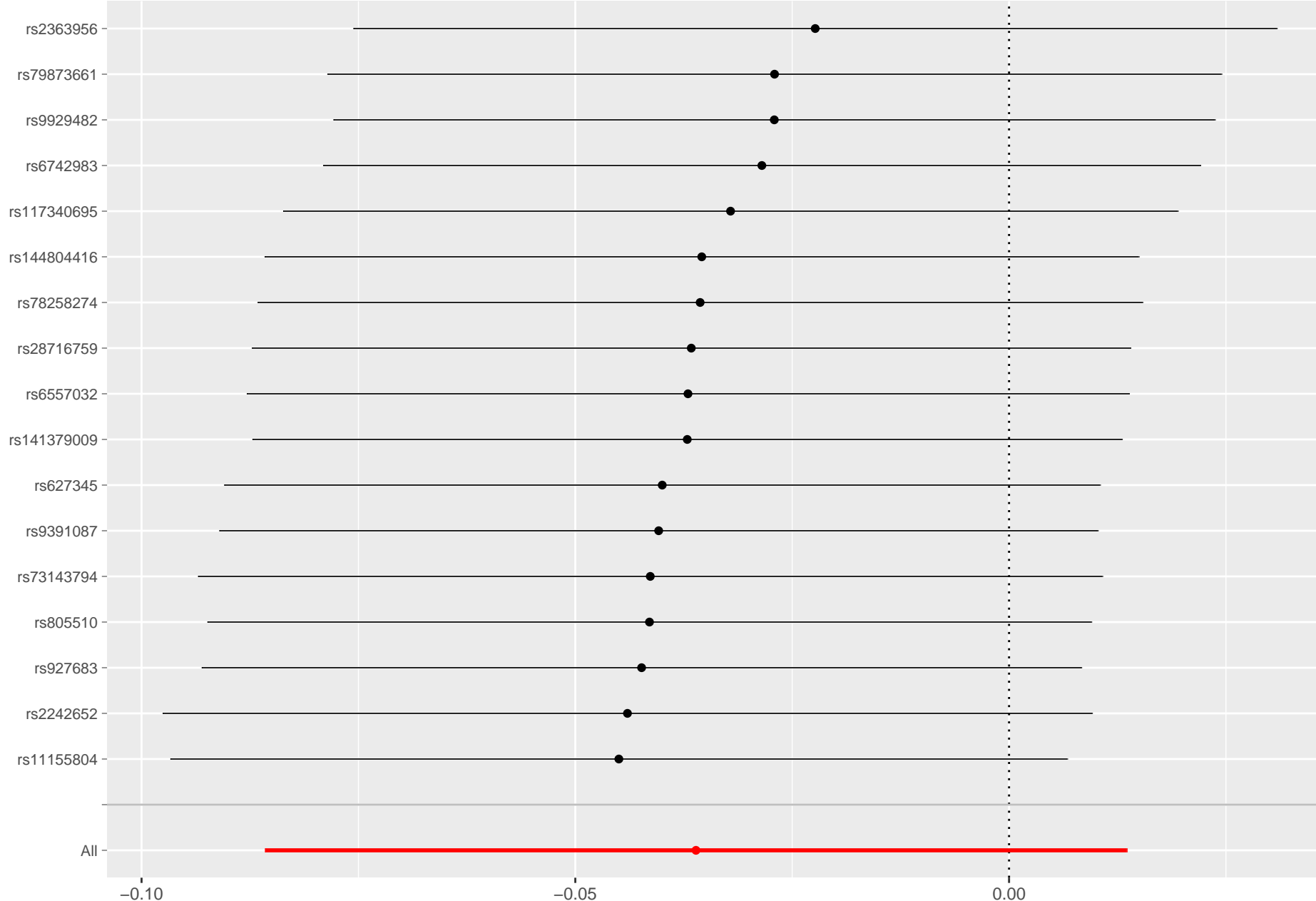

Supplement: Supplementary file 1 — Supporting Information Additional supporting information can be found online in the Supporting Information section. [file IJE-2026-7371037-s001.zip › Figure S2B.pdf]

# MR Method

- Inverse variance weighted
- MR Egger

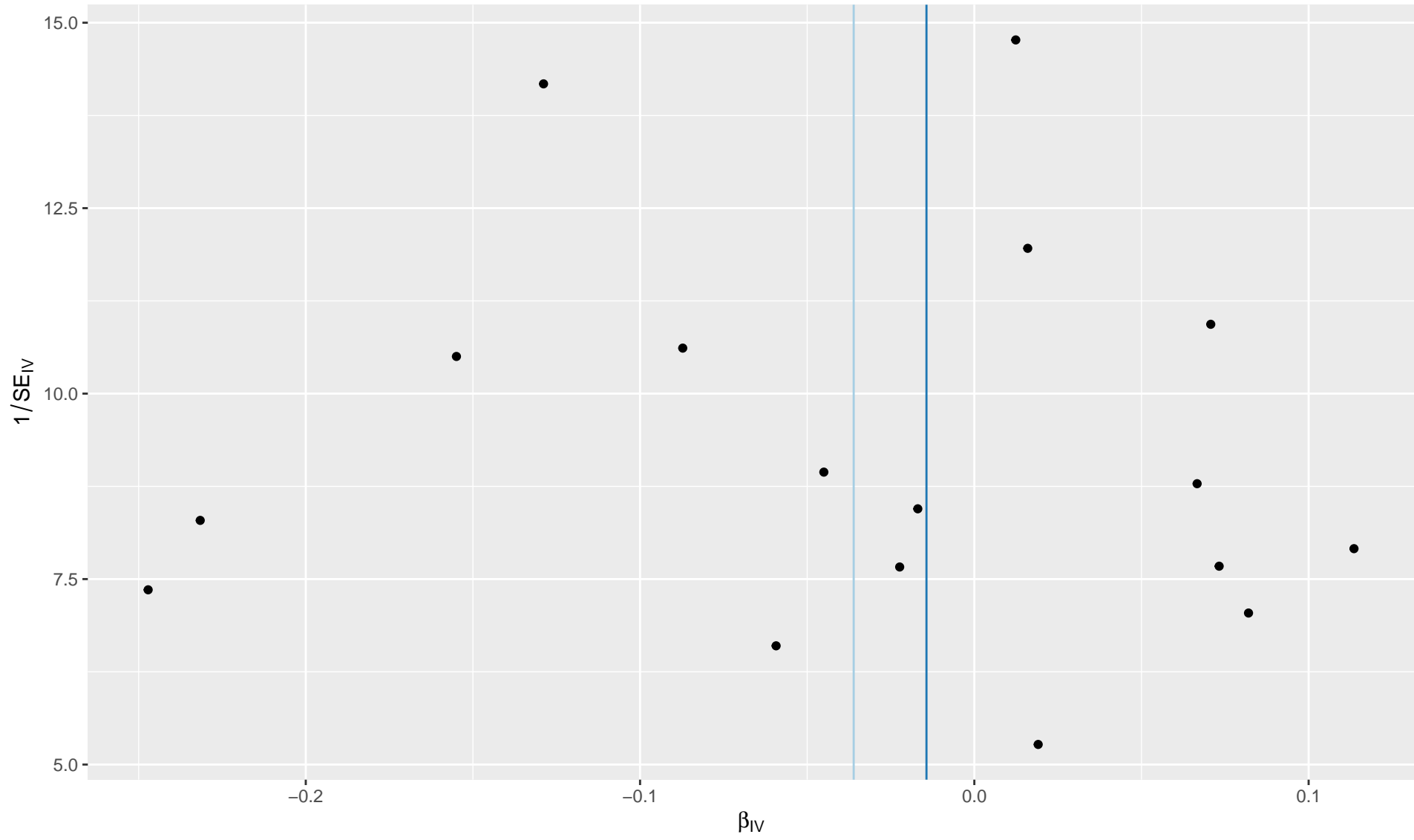

Supplement: Supplementary file 1 — Supporting Information Additional supporting information can be found online in the Supporting Information section. [file IJE-2026-7371037-s001.zip › Figure S2D.pdf]

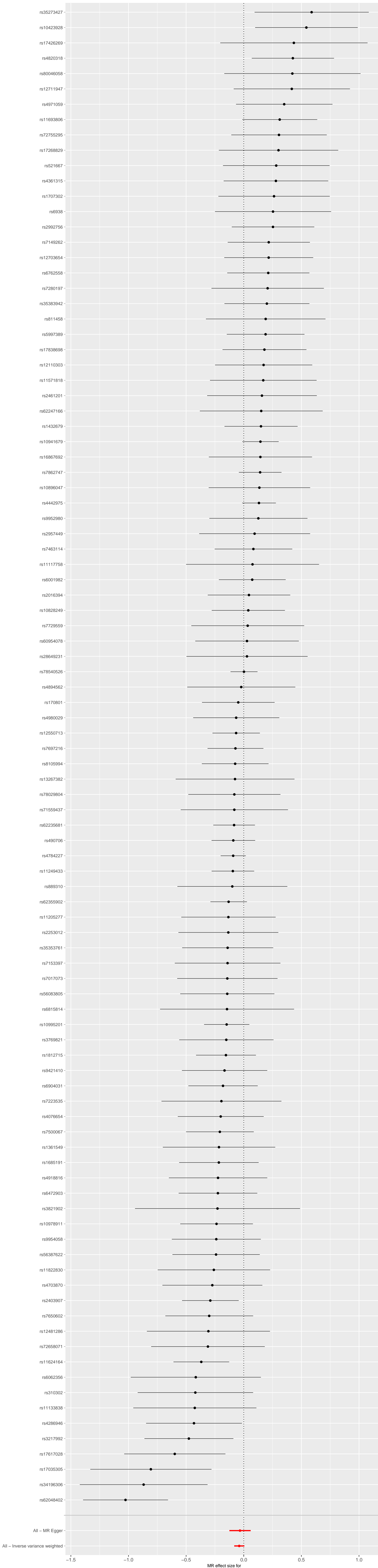

Supplement: Supplementary file 1 — Supporting Information Additional supporting information can be found online in the Supporting Information section. [file IJE-2026-7371037-s001.zip › Figure S3A.pdf]

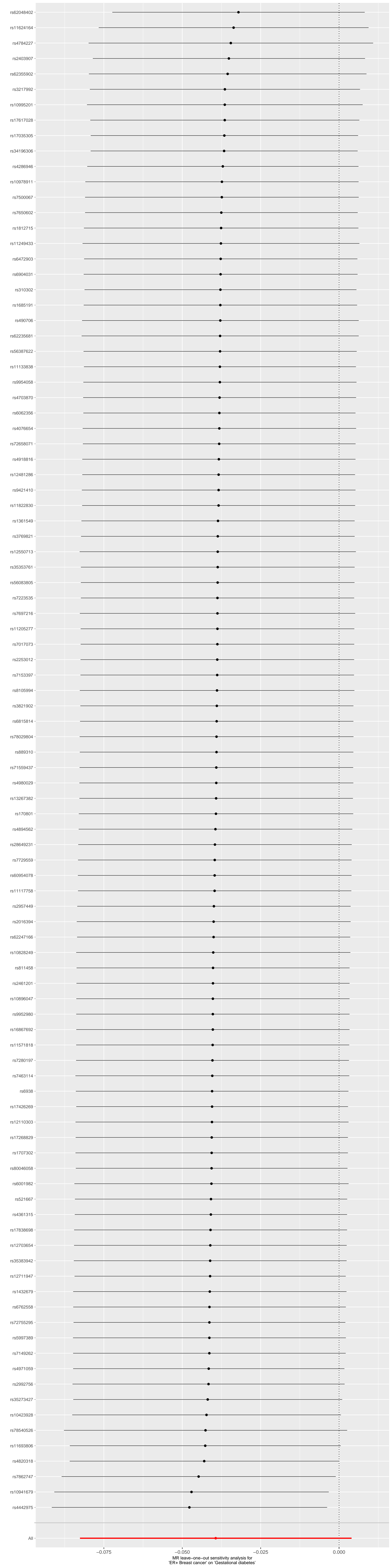

Supplement: Supplementary file 1 — Supporting Information Additional supporting information can be found online in the Supporting Information section. [file IJE-2026-7371037-s001.zip › Figure S3B.pdf]

# MR Method

- Inverse variance weighted
- MR Egger

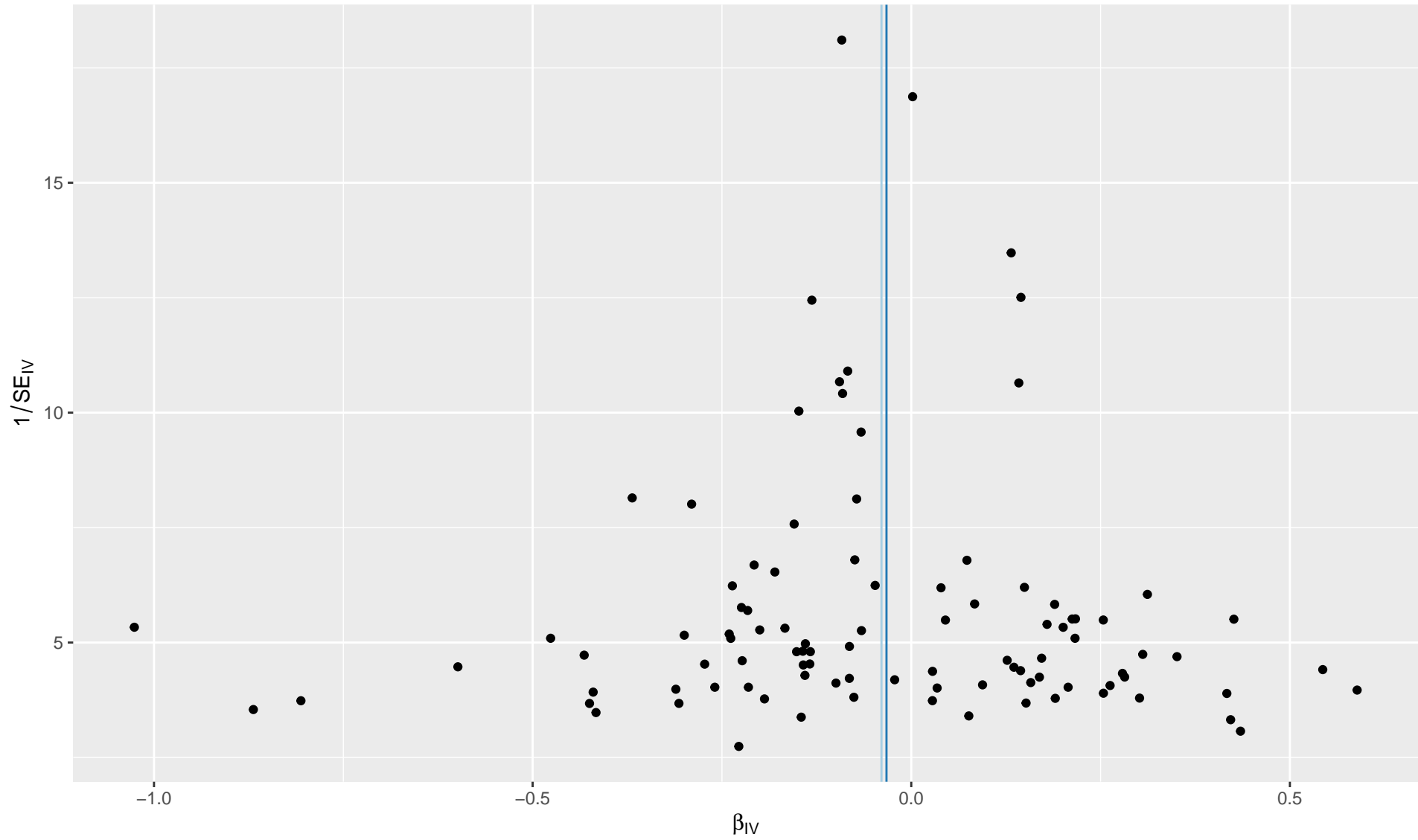

Supplement: Supplementary file 1 — Supporting Information Additional supporting information can be found online in the Supporting Information section. [file IJE-2026-7371037-s001.zip › Figure S3D.pdf]
