# Supplementary material for: Genetic Evidence Links Gestational Diabetes Mellitus to Increased ER‐Negative Breast Cancer Risk Through a Mendelian Randomization Analysis
Source: Int J Endocrinol. 2026 Apr 28;2026:7371037. doi: 10.1155/ije/7371037 (PMC13122568; doi:10.1155/ije/7371037)
Supplement: Supplementary file 1 — Supporting Information Additional supporting information can be found online in the Supporting Information section. [file Figuer S2C.pdf › Figuer S2C.pdf]

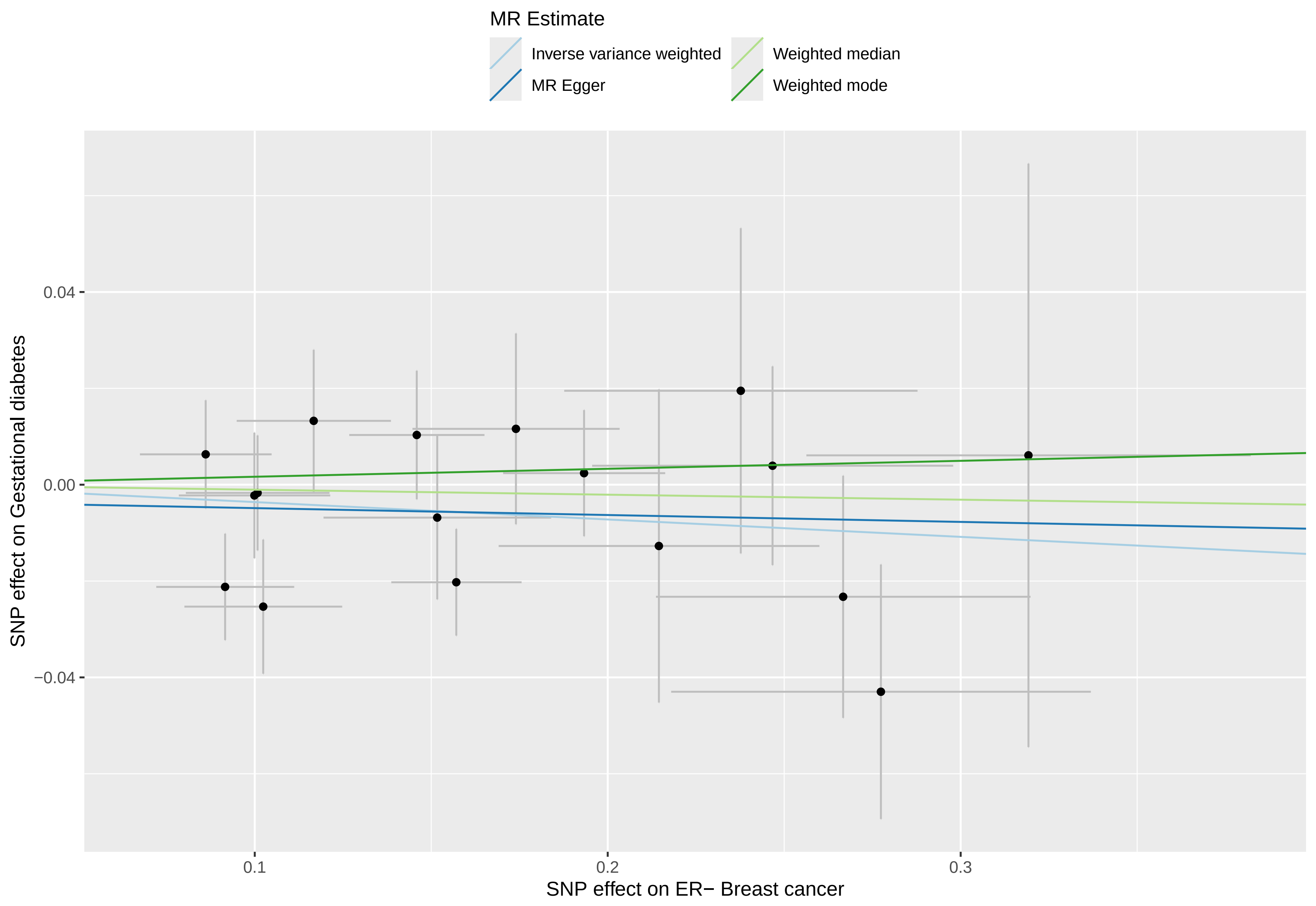

MR Estimate
Inverse variance weighted
MR Egger
Weighted median
Weighted mode
SNP effect on Gestational diabetes
0.04
0.00
−0.04
0.1
0.2
0.3
SNP effect on ER− Breast cancer

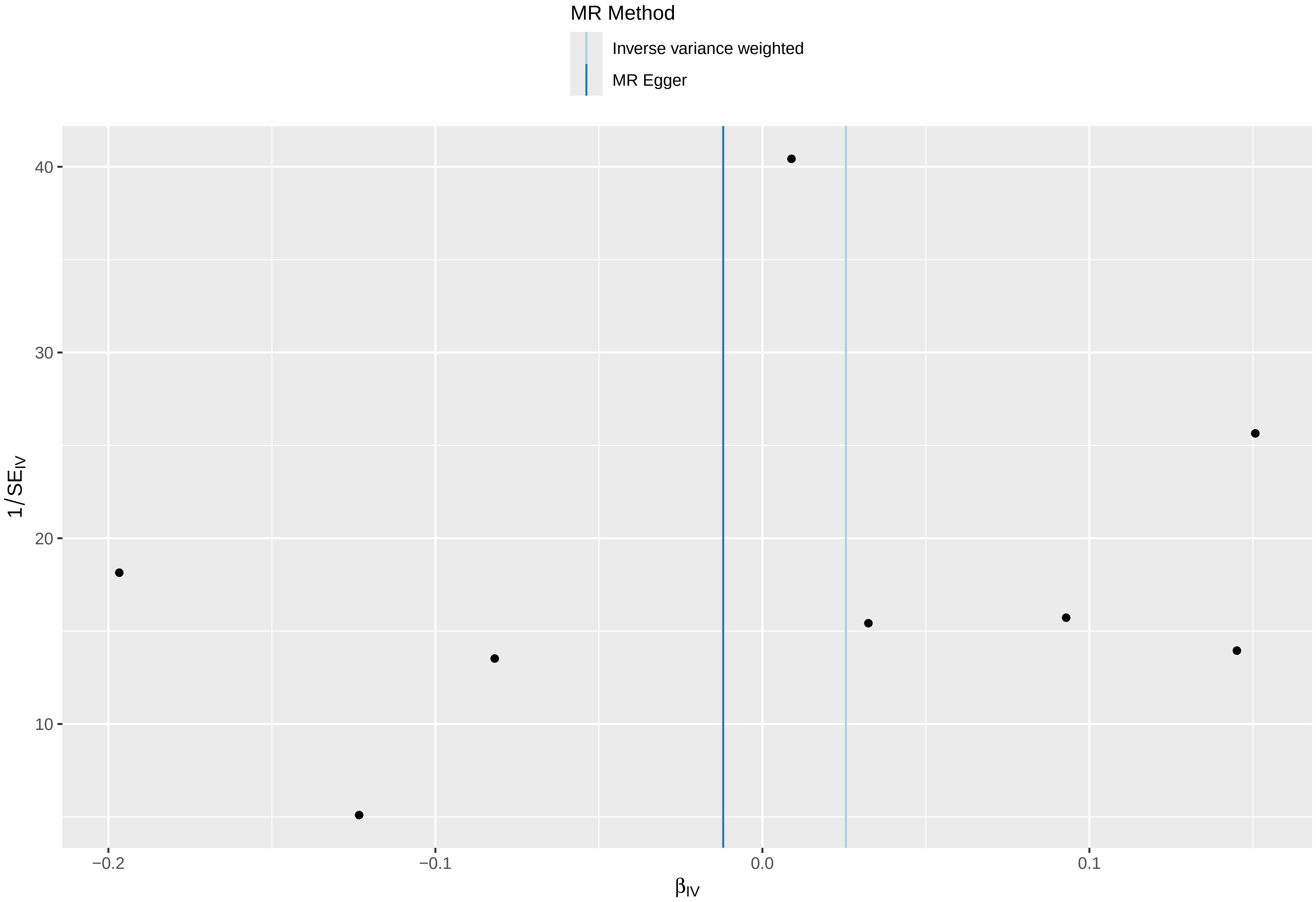
MR Method
Inverse variance weighted
MR Egger
40
30
20
10
1/SE$_{IV}$
−0.2
−0.1
0.0
0.1
β_{IV}

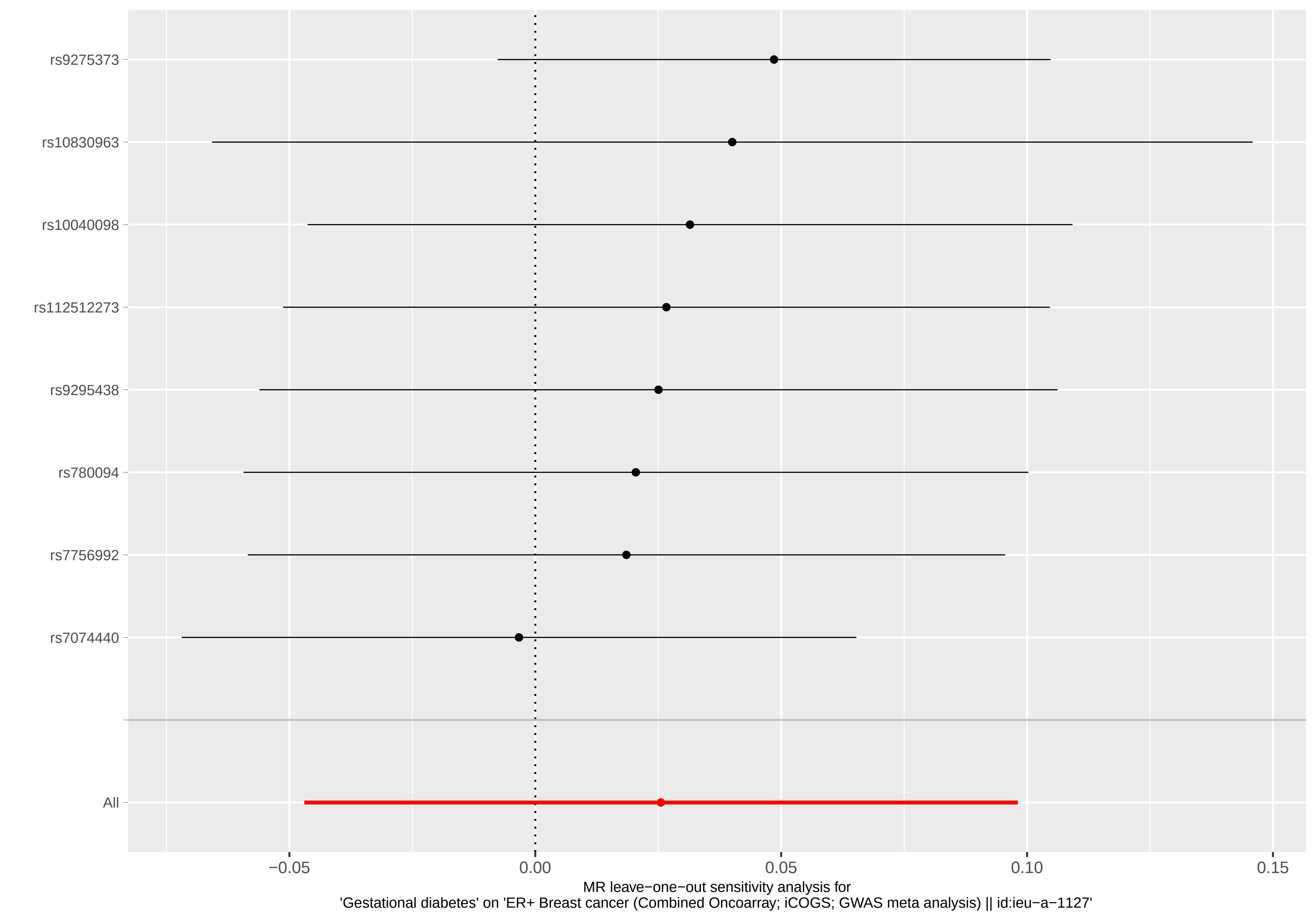

rs9275373
rs10830963
rs10040098
rs112512273
rs9295438
rs780094
rs7756992
rs7074440
All
−0.05
0.00
0.05
0.10
0.15
MR leave−one−out sensitivity analysis for
'Gestational diabetes' on 'ER+ Breast cancer (Combined Oncoarray; iCOGS; GWAS meta analysis) || id:ieu−a−1127'
